# Restoration of UAV-Based Backlit Images for Geological Mapping of a High-Steep Slope

**DOI:** 10.3390/s24051586

**Published:** 2024-02-29

**Authors:** Tengyue Li

**Affiliations:** 1 Key Laboratory of Geophysical Exploration Equipment Ministry of Education of China, Jilin University, 938 West Democracy Street, Changchun 130026, China; litengyue@jlu.edu.cn; Tel.: +86-15318866927; 2 College of Construction Engineering, Jilin University, 938 West Democracy Street, Changchun 130026, China; 3 Badong National Observation and Research Station of Geohazards, China University of Geosciences, Wuhan 430074, China

**Keywords:** geological mapping, backlit image, image restoration, UAV

## Abstract

Unmanned aerial vehicle (UAV)-based geological mapping is significant for understanding the geological structure in the high-steep slopes, but the images obtained in these areas are inevitably influenced by the backlit effect because of the undulating terrain and the viewpoint change of the camera mounted on the UAV. To handle this concern, a novel backlit image restoration method is proposed that takes the real-world application into account and addresses the color distortion issue existing in backlit images captured in high-steep slope scenes. Specifically, there are two main steps in the proposed method, which consist of the backlit removal and the color and detail enhancement. The backlit removal first eliminates the backlit effect using the Retinex strategy, and then the color and detail enhancement step improves the image color and sharpness. The author designs extensive comparison experiments from multiple angles and applies the proposed method to different engineering applications. The experimental results show that the proposed method has potential compared to other main-stream methods both in qualitative visual effects and universal quantitative evaluation metrics. The backlit images processed by the proposed method are significantly improved by the process of feature key point matching, which is very conducive to the fine construction of 3D geological models of the high-steep slope.

## 1. Introduction

**Figure 1:**
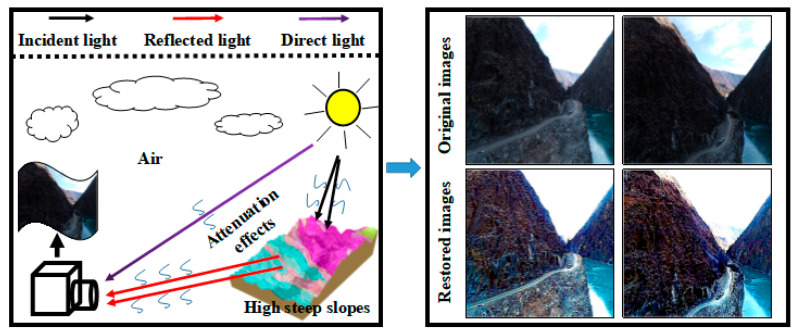
The atmospheric imaging model (on the **left**) and the outcomes of the suggested method for restoring degraded backlit images (on the **right**).

Unmanned aerial vehicle (UAV)-based explorations are now widely used in the field of geological investigations in high-steep slope scenes, which can provide precious visual perception data and support the subsequent analysis of 3D geological models [1]. However, the images are usually captured under sub-optimal lighting conditions when the UAV is moving and shooting undulating and unbroken high-steep slope scenes. Due to the inevitable environmental constraints, poor intelligibility of details caused by the backlit effect has become one of the most common image degradation issues in backlit images [2,3]. When the light source is positioned directly across from the camera, a significant portion of the light emanating from it is funneled directly into the camera lens. This leads to highly disparate exposure levels between the shadowy foreground and the well-lit background [4]. Images captured with a backlit setup exhibit minimal contrast, and the image details are scarcely discernible (as illustrated in Figure 1). This severely hampers the effectiveness of advanced computer vision tasks like object recognition and 3D reconstruction. The pronounced backlit effects within the images result in the breakdown of object recognition and 3D reconstruction tasks. Consequently, the restoration of these compromised backlit images holds considerable significance for a wide range of vision-based applications.

The degraded backlit image problem is a typical image inverse problem that is related to uneven illumination and low-light imaging. Current approaches [5,6,7,8] primarily endeavor to recover backlit images by augmenting their contrast through methods such as histogram equalization (HE), contrast-limited adaptive histogram equalization (CLAHE), and Retinex-based algorithms. Moreover, experimental images have predominantly focused on visual imaging in close proximity. These degraded backlit images can be easily refined using edge-aware tone mapping or illumination layer separation. However, there is little literature to address this tricky issue for real-world engineering geology applications, especially for applications related to UAV-based backlit image restoration in high-steep slope scenes. These images simultaneously suffer from the backlit effect and the color distortion issue caused by UAV movement and long-range imaging, resulting in deteriorated visual image quality.

This paper introduces an innovative approach for restoring low-light images captured by UAVs in high-steep slope environments. These degraded images suffer from the low contrast issue caused by the backlit effect and the color distortion issue caused by the atmosphere’s physical imaging attenuation. To address the issues of low contrast and color distortion, we build a backlit image formation model that considers both the backlit effect and light attenuation. We remove the backlit effect using the Retinex theory model and improve the color and sharpness using a color and detail enhancement model. The experimental findings affirm the benefits of the proposed method for restoring degraded backlit images, surpassing the performance of other contemporary approaches.

The key contributions of this paper can be succinctly outlined as follows: (1) We introduce an innovative model for backlit image formation, which offers a compelling physical explanation for the generation of degraded backlit images in high-steep slope scenarios; (2) We propose a backlit image restoration method that leverages the principles of the Retinex theory and the physical image formation model to mitigate the impact of backlit and enhance the overall color representation of the image.

The remainder of this paper is structured as follows: Section 2 provides a summary of related works. Section 3 elaborates on the proposed method for restoring degraded backlit images. Section 4 presents and discusses the experimental results, while Section 5 serves as the conclusion of this paper.

## 2. Related Works

Numerous researchers have put forward diverse image restoration algorithms for the enhancement of low-light images, including degraded backlit images. These algorithms can be categorized broadly into histogram equalization (HE)-based techniques [5,6,9,10,11,12,13,14,15], Retinex-based methods [7,8,16,17,18,19,20,21,22,23,24,25,26], deep learning-based approaches [27,28,29,30,31,32,33,34,35], and hybrid methods [36,37,38,39]. HE-based methods and their variations have been the subject of extensive study for restoring low-light images over the past few decades. These techniques aim to enhance low-light images by expanding the dynamic range of observed images. For instance, CLAHE, a pioneering HE method, was initially developed to display intensity levels in medical images and has demonstrated competitive performance [5]. Ibrahim et al. [9] introduced the brightness-preserving dynamic histogram equalization (BPDHE) as an extension of HE. BPDHE can generate images with a mean intensity nearly equal to that of the original image while maintaining overall brightness. Celik et al. [12] further advanced HE by incorporating a 2D histogram and considering the relationships between each pixel and its neighboring pixels for low-light image restoration. Experimental results indicate that Celik et al.’s method [12] produces satisfactory enhancements. Shi et al. [6] proposed a normalized gamma transformation CLAHE with color correction in the lab color space. They conducted extensive experiments on low-light images, yielding competitive results. However, HE-based methods and their variants primarily focus on enhancing image contrast rather than image illumination. These methods also struggle to remove the intense noise present in low-light images. In recent years, Retinex-based methods have gained attention. Fu et al. [7] introduced a probabilistic image enhancement method based on the simultaneous estimation of illumination and reflectance in the linear domain, achieving comparable results in both subjective and objective assessments. They later proposed a weighted variational model for estimating both reflectance and illumination, extending their Retinex-based approach [19]. Li et al. [23] devised an optimization function based on the robust Retinex model, featuring regularization terms for illumination and reflectance. They extended this method to underwater image enhancement and remote sensing applications. Ren et al. [26] presented a robust low-light enhancement technique, incorporating a low-rank prior into a Retinex decomposition to suppress noise in the reflectance map. This method excels in both image enhancement and denoising. However, Retinex-based methods, while improving the visibility of dark areas, can also amplify intense noise due to inaccurate reflectance estimation. Deep learning-based methods provide efficient end-to-end solutions and achieve high performance owing to their powerful feature representation capabilities. Lv et al. [27] introduced a multi-branch low-light enhancement network capable of extracting rich features from different levels and generating output images via multi-branch fusion. This approach holds significant potential for enhancing both low-light images and videos. Li et al. [28] proposed a trainable CNN for addressing weakly illuminated images. They leveraged a Retinex-based model and an illumination map generated from a deep learning-based network to restore images. Wang et al. [34] introduced a normalizing flow network that treats low-light features as conditions and learns to map the distribution of normally exposed images into a Gaussian distribution, performing well in restoring illumination and color. Wu et al. [35] presented a Retinex-based deep unfolding network that integrates an optimization strategy into the layers’ decomposition of reflectance and illumination. They claim that this method can preserve details and suppress noise in the final results. However, deep learning-based methods occasionally produce unnatural results due to a lack of modeling of the formation of natural low-light images. In the realm of unified methods, two primary approaches are prominent: the camera response model and fusion-based framework [36,37] and the joint illumination and denoising framework [38,39]. The former combines a dual-exposure fusion method and a camera response model to enhance contrast and brightness [36,37]. The latter first enhances input images and then performs denoising operations [38,39]. These methods can yield excellent results, but they lack a robust physical explanation. Furthermore, in recent years, several underwater image enhancement methods [40,41] have demonstrated potential in restoring low-contrast images. These methods have reported generalizability to land-based low-light images.

Existing literature has reported inspiring results. However, these methods handle degraded backlit or low-light images captured in a very close range, and they show limited ability to address real-world applications such as degraded backlit images captured in high-steep slope scenes using UAVs. Aside from the backlit effect, these images also suffer from color distortion when light propagates through the atmosphere. Moreover, the mountains usually have a single color, and it is helpful to enhance the color and improve the sharpness for the subsequent 3D geological modeling and structural surface identification. To the best of my understanding, no existing method has been capable of simultaneously addressing both the backlit effect and the color distortion in images captured in high-steep slope settings. I endeavor to tackle these dual challenges in this paper.

## 3. Proposed Method

### 3.1. Backlit Image Formation Model

In terms of the backlit image formation model, the Retinex theory [42] is often used to address this inverse problem [8,23]. Drawing upon the Retinex theory, the observed image *I*_1_(*x*) is considered the result of the interaction between the reflectance *R*(*x*) and the illumination *F*(*x*) of the image. This relationship can be formulated as

(1) $$I_{1}(x)=R(x)\cdot F(x),$$

where *x* represents a pixel in the image. Later, it was proved that intensive noise should be an inevitable term in real-world applications [23]; thus, it can be expressed as

(2) $$I_{2}(x)=R(x)\cdot F(x)+N(x),$$

where *N*(*x*) denotes the intensive noise. UAVs usually maintain a safe distance of tens or hundreds of meters from complex object scenes when capturing images of high-steep slopes. In this process, the light coming into the on-board camera is attenuated when traveling through the air [43]. As the intensive noise is an important item, we define it as *N_A_*(*x*), which introduces an additive component to the image that directly reduces the image contrast and visibility. Other items (e.g., errors introduced by camera sensors or motion blur) are defined as *N*_0_(*x*). Therefore, the intensive noise can be defined as

(3) $$N(x)=N_{A}(x)+N_{0}(x).$$

As for *N_A_*(*x*), it represents the propagation of light in the atmosphere. The degradation of the image resulting from *N_A_*(*x*) affects each pixel in the image. It relies on the distance between the object scene and the camera, which can be articulated as follows:

(4) $$N_{A}(x)=t(x)\cdot R(x)+(1-t(x))\cdot A,$$

(5) $$t(x)=e^{-\beta d(x)},$$

where *t*(*x*) represents the scene transmission, *d*(*x*) represents the object scene distance at pixel *x*, *β* denotes the atmospheric attenuation coefficient, and *A* is the air light in the image areas that represents a single color [43]. Thus, we regard the observed image *I*_3_(*x*) as

(6) $$I_{3}(x)=[R(x)\cdot F(x)+N_{0}(x)]+[t(x)\cdot R(x)+(1-t(x))\cdot A].$$

There are two items in Equation (6); the first item [.] comes from the Retinex theory model, and the second item [.] comes from the physical imaging formation model. Therefore, the observed image *I*_3_(*x*) is the simultaneous atmospheric light attenuation that occurs in a backlit shooting situation. To restore the degraded backlit images, we consider a combination of backlit removal and compensation for atmospheric light attenuation. We obtained experience from the previous works [38,39] and performed two-stage operations to restore the degraded backlit images, which combined the realities of shooting the high-steep slope scenes using UAVs. We first applied the backlit removal algorithm to restore the backlit images and complete the initial restoration. The initial results are similar to the images captured by normal cameras with good light conditions. However, they still suffer from color degradation and slight haze when shooting high-steep slopes from a distance using UAVs. Hence, we utilize the haze-line technique to further improve the color and detail of the initial results. Severe degraded backlit images can be effectively restored by backlit removal and the haze-line technique.

### 3.2. Backlit Removal

We used the wisdom from the previous works [7,21,23] to perform the backlit removal. According to Fu et al.’s research [7], the estimation problem of the reflectance and illumination can be transformed into an objective function minimization problem. We defined the objective function as *E*(*F*, *R*)

(7) $$E(F,R)={\left\|R\cdot F-I\right\|}_{F}^{2}+\alpha {\left\|\nabla F\right\|}_{1}+w{\left\|\nabla R-K\cdot \nabla I\right\|}_{F}^{2},$$

(8) $$K=1+\lambda \cdot e^{-\left|\nabla I\right|/\sigma },$$

where ${\left\|\;\cdot \;\right\|}_{F}$ is the Frobenius norm, ${\left\|\;\cdot \;\right\|}_{1}$ is the l1 norm, and *α* and *w* are the parameters of ${\left\|\nabla F\right\|}_{1}$ and ${\left\|\nabla R-K\cdot \nabla I\right\|}_{F}^{2}$, respectively. K⋅∇I represents the gradient of the observed image *I*, which can be adjusted. In Equation (7), ${\left\|R\cdot F-I\right\|}_{F}^{2}$ constrains the fidelity and minimizes the gap between the estimated R⋅F and the observed image *I*, ${\left\|\nabla F\right\|}_{1}$ imposes smoothness on the illumination map *F*, and ${\left\|\nabla R-K\cdot \nabla I\right\|}_{F}^{2}$ strengthens the structural details of the reflectance by minimizing the disparity between the gradient of the reflectance *R* and *I*. According to Equations (2), (6), and (7), the decomposition model considers a practical application of Retinex theory and can be expressed as

(9) $$E(F,R,N)={\left\|R\cdot F+N-I\right\|}_{F}^{2}+\alpha {\left\|\nabla F\right\|}_{1}+w{\left\|\nabla R-K\cdot \nabla I\right\|}_{F}^{2}+\lambda {\left\|N\right\|}_{F}^{2},$$

where *N* represents the noise map and $\lambda {\left\|N\right\|}_{F}^{2}$ represents the constraint on the overall noise intensity. We achieved the local minima of *E*(*F*, *R*, *N*) using the ADMM theory [7,23,44] and obtained the output *R*_1_ after the backlit removal step.

### 3.3. Color and Detail Enhancement

The backlit effect in captured images was removed and we obtained the backlit-free results after backlit removal. However, the images still suffered from color degradation and slight haze that the backlit removal strategy could not handle. To restore the color and improve the sharpness, we followed the previous wisdom of Li et al.’s research [45] and proceeded to take an additional step in order to enhance the color and finer details within the image content. In this subsection, the observed image is regarded as *R*_1_(*x*), and we define the atmospheric image *I_A_*(*x*) as

(10) $$I_{A}(x)=R_{1}(x)-A.$$

From Equation (4), we can infer that the atmospheric image *I_A_*(*x*) is able to be expressed as

(11) $$I_{A}(x)=(R(x)-A)\cdot t(x).$$

To estimate the parameters conveniently, we transfer the equation into spherical coordinates, which is defined as

(12) $$I_{A}(x)=[r(x),Lat(x),\mathrm{Long}(x)],$$

(13) $$r(x)=t(x)\left\|R(x)-A\right\|,\;0\leq t(x)\leq 1,$$

where *r*(*x*) represents the distance from the background light source to the pixel, while *Lat*(*x*) and *Long*(*x*) denote the latitude and the longitude, respectively. *r*(*x*) reaches its maximum value when *t*(*x*) is set to 1; at this point, we define the transmission as

(14) $$t(x)=\frac{r(x)}{r_{\max}}.$$

Based on the pixel assumption of the haze-line [43], haze-free pixels are present along a haze-line *H* that meets ${\hat{r}}_{\max}(x)=\underset{x\in H}{\max}\{r(x)\}$. Then, the revised transmission ${\hat{r}}_{\max}(x)=\underset{x\in H}{\max}\{r(x)\}$ is expressed as

(15) $$\hat{t}(x)=\frac{r(x)}{{\hat{r}}_{\max}(x)}.$$

As *R* is a positive term, the transmission has a minimum limit, which is defined as

(16) $$t_{lb}(x)=\left\{\begin{array}{c} 1-\min\{\frac{R_{1}^{r}(x)}{A_{r}(x)}\}\\ 1-\min\{\frac{R_{1}^{g}(x)}{A_{g}(x)}\}\\ 1-\min\{\frac{R_{1}^{b}(x)}{A_{b}(x)}\} \end{array}\right.,$$

where *r*, *g*, and *b* represent the red, green, and blue color channels, respectively. Based on the above analysis, the minimum limit of the transmission is revised as

(17) $${\hat{t}}_{lb}(x)=\max\{\hat{t}(x),t_{lb}(x)\}.$$

Then, the regularization optimization method is used to address the initial transmission estimation problem to reduce estimation errors, and the formula can be expressed as

(18) $$\min\left\{\sum_{x}\frac{{\left[\hat{t}(x)-{\hat{t}}_{lb}(x)\right]}^{2}}{\xi^{2}(x)}+\psi \cdot \sum_{x}\sum_{y\in N_{bx}}\frac{{\left[\hat{t}(x)-\hat{t}(y)\right]}^{2}}{{\left\|R_{1}(x)-R_{1}(y)\right\|}^{2}}\right\},$$

where ξ(x) denotes the standard deviation of ${\hat{t}}_{lb}(x)$, ψ represents the parameter for fine-tuning the function’s balance, and $N_{bx}$ refers to the adjacent pixels. In terms of Equation (17), the initial and subsequent terms correspond to the data and smoothing components, respectively. The data component, denoted as ${\hat{t}}_{lb}(x)$, relies on the standard deviation to ensure stable transmission estimation and prevent significant deviations. Meanwhile, the smoothing term effectively eliminates noise from neighboring image blocks, thereby enhancing the preservation of critical edges and details while improving overall image smoothness. In the end, we derive the restored image $\hat{R}$ by employing the transmission values $\hat{t}(x)$ and A through Equation (18):

(19) $$\hat{R}(x)=\frac{\left\{R_{1}(x)-\left[1-\hat{t}(x)\right]\cdot A\right\}}{\hat{t}(x)}$$

### 3.4. Selected Comparison Techniques and Environment Settings

To showcase the efficacy of the proposed approach, we carried out comprehensive evaluations on real-world UAV-based degraded backlit images in high-steep slope scenes. We chose several state-of-the-art degraded image restoration techniques for comparison, including the methods of Li et al. [40], Fu et al. [7], Wang et al. [8], Shi et al. [6], Zhang et al. [41], Ying et al. [36], Lv et al. [27], and Wu et al. [35]. Among them, the conventional methods of Li et al. [40], Fu et al. [7], Wang et al. [8], Shi et al. [6], Zhang et al. [41], and Ying et al. [36] mainly focus on removing uneven illumination, separating the illumination layer, or improving the contrast. The techniques presented by Lv et al. [27] and Wu et al. [35] are representative CNN-based algorithms designed to enhance low-light images through feature map manipulation. Additionally, the methods introduced by Li et al. [40], Fu et al. [7], Wang et al. [8], Shi et al. [6], Zhang et al. [41], and Ying et al. [36] and the proposed approach were executed on a personal computer running the Windows 10 operating system, equipped with an Intel i7-9700K CPU (Santa Clara, CA, USA) clocked at 3.60 GHz. The source code for the proposed method was implemented using Matlab [46]. Lv et al.’s method [27] and Wu et al.’s method [35] were executed on the Ubuntu 16.04 platform, utilizing an Intel i7-9700K CPU and Nvidia 1070Ti GPU (Santa Clara, CA, USA). The source code for these methods was developed using the Keras [47] and PyTorch [48] platforms, respectively. All code executions of the comparison methods are publicly available on Github from their research works, and they were run with the authors’ default parameters. Several quantitative contrast evaluation metrics, including CEIQ [49], PCQI [50], and UCIQE [51], were chosen for comparing the experimental results. CEIQ is a no-reference quality assessment metric that assesses contrast in distorted images by leveraging natural scene statistics principles. PCQI is a metric based on an adaptive representation of local patch structure, enabling precise predictions of human perception regarding contrast variations. In the case of CEIQ and PCQI metrics, higher values are preferable. UCIQE is designed to quantify non-uniform color cast, blurring, and low contrast in images, and a higher UCIQE value suggests that the image exhibits a better equilibrium among chroma, saturation, and contrast.

### 3.5. Data Collection

**Figure 2:**
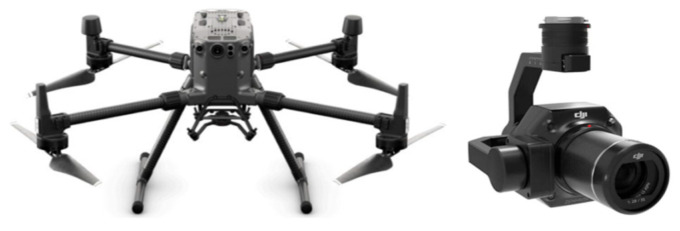
The UAV platform (**left**) and the on-board camera (**right**).

**Figure 3:**
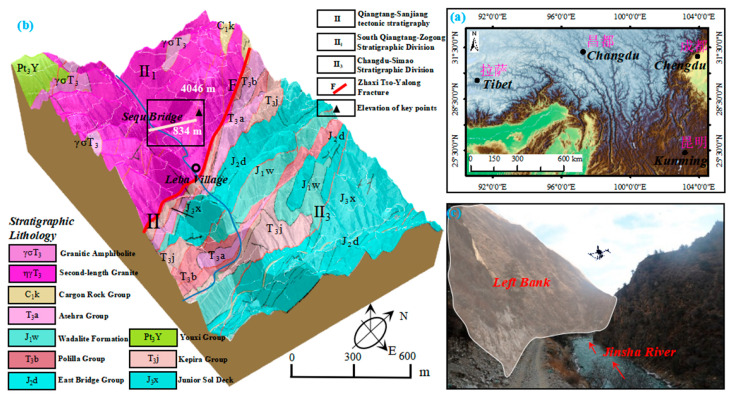
(**a**) The geographical location of the study area; (**b**) The geological and structural map; (**c**) The experimental scene of the high-steep slope.

**Figure 4:**
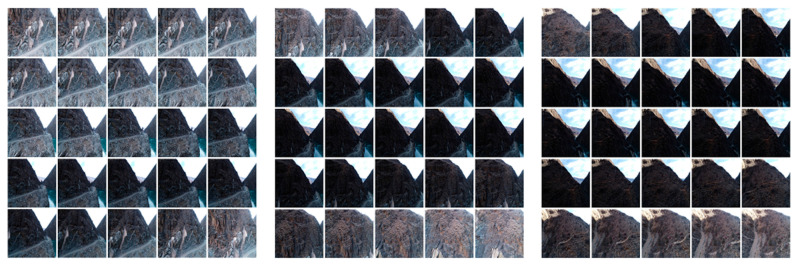
Three cases of backlit images extracted from the dataset; they are the examples of cases 1, 2, and 3 (from left to right), respectively.

We used DJI M300 RTK UAV (Shenzhen, China) to perform the terrain exploration. The UAV boasts a maximum operational altitude of 5000 m and is equipped with a six-way obstacle avoidance system, making it suitable for close operations in intricate environments. The Zenith P1 35 mm fixed-focus full-frame high-resolution camera was installed on the DJI M300 RTK UAV, which is able to capture high-quality images. Figure 2 illustrates the DJI M300 RTK UAV and its onboard camera, and the corresponding specific parameters are shown in Table 1. We collected the data using UAV visual flight detection in Sequ Bridge, Changdu City, Tibet Autonomous Region; the data collection sites and locations are shown in Figure 3. The study area is an alpine valley landscape with large topographic relief and a deep river valley. The valley of the Sequ River is a “V”-shaped valley with steep slopes on both sides. We executed a total of five flights, which covered about 0.12 km^2^. The overlap of two adjacent images is 85% and 70% in the heading and side directions, respectively. The UAV was set to a speed of 1.5 m/s with a shooting interval of two seconds, and we finally acquired 745 images with a resolution of 8192 × 5460 pixels. We selected three backlit cases from the captured images (shown in Figure 4), which demonstrate the backlit effect created by the angle change of the UAV when operating on the high-steep slope. Thirty severely backlit images were selected as experimental data in the ablation study and comparison experiments. To improve the computational efficiency, the images were all resized to 512 × 512.

## 4. Experimental Results and Analysis

The proposed framework integrates a Retinex-based model for removing the backlit and a physical imaging formation model for enhancing the color and the details. We performed ablation experiments to assess the efficacy of each model, utilizing a dataset consisting of 30 backlit images sourced from three distinct high-steep slope scenes, with 10 images per scene.

### 4.1. Ablation Study

**Figure 5:**
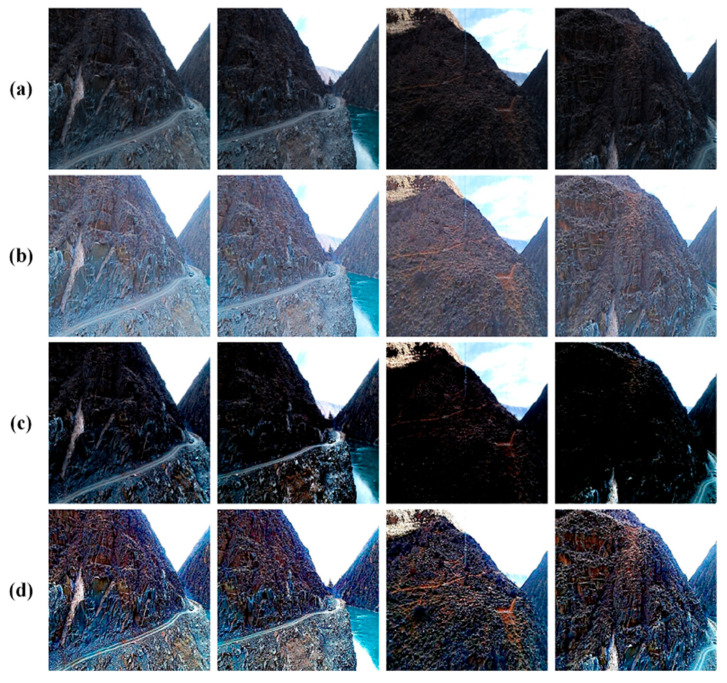
Qualitative outcomes from the ablation study. (**a**) The input images; (**b**) The method of backlit removal; (**c**) The method of color and detail enhancement; (**d**) The proposed method.

The ablation experiments’ qualitative comparisons can be seen in Figure 5, while the quantitative comparison results are displayed in Table 2. Based on Figure 5, it is evident that the proposed method produces visual results that closely resemble real-world images in terms of natural color and contrast. The Retinex-based model without the physical imaging formation branch achieves good performance in removing the backlit and improving the contrast; however, the haze effect remains in the resultant images. As for the color and detail enhancement model, it improves monotonous and dull images into vivid ones, but the model presents very limited capability for addressing the backlit effect. We also conducted a comparison between two models: the Retinex-based model and the model for enhancing color and detail. The former model performs much better in contrast enhancement so that more global content can be observed, but it also introduces a misty veil. The latter one has a better visual impact in color and sharpness enhancement; however, it contributes little to the contrast enhancement. When we integrate the Retinex-based model and the color and detail enhancement branch, we achieve improved brightness, contrast, and color enhancement simultaneously. The quantitative results are presented in Table 2, and from these outcomes, we can infer that the quantitative findings align with the qualitative ones. The proposed method secures the top position in two out of three evaluation metrics (CEIQ and PCQI), with a slight margin separating its results from those obtained using backlit removal in the UCIQE metric. These pieces of evidence indicate that the proposed method combines the strengths of both the Retinex-based model and the physical imaging formation model.

### 4.2. Qualitative Evaluation

**Figure 6:**
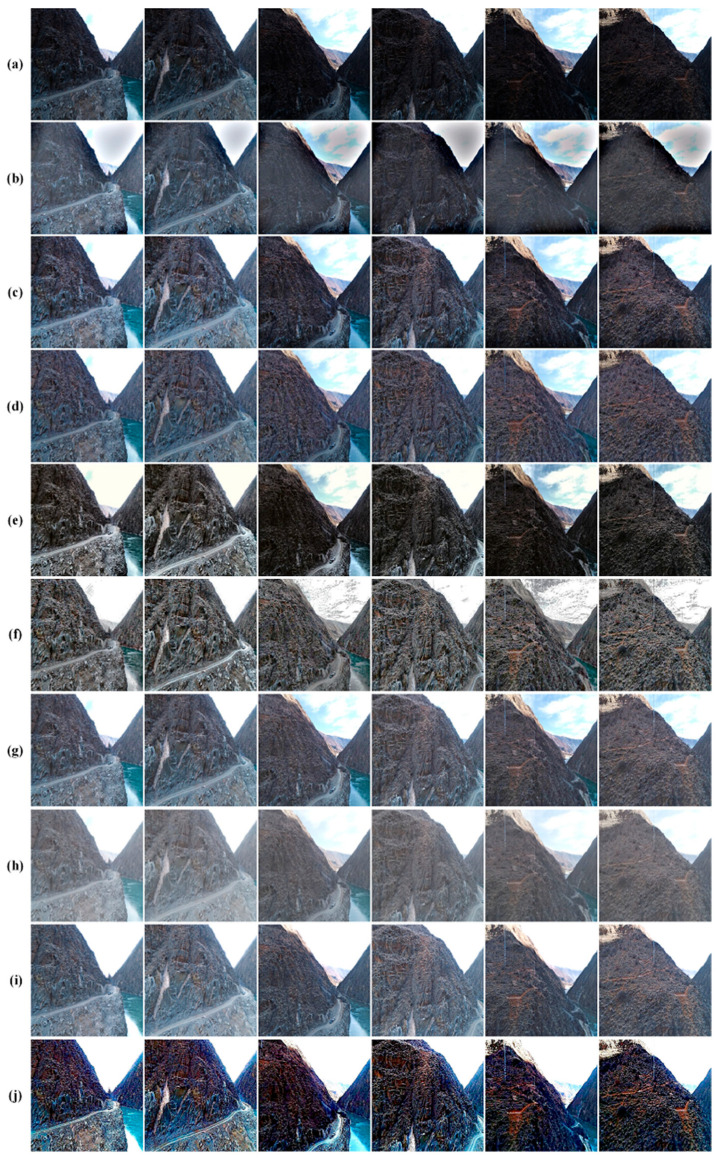
The qualitative assessment outcomes of backlit images captured in scenes with high-steep slopes. (**a**) The input images; (**b**) The method of Li et al. [40]; (**c**) The method of Fu et al. [7]; (**d**) The method of Wang et al. [8]; (**e**) The method of Shi et al. [6]; (**f**) The method of Zhang et al. [41]; (**g**) The method of Ying et al. [36]; (**h**) The method of Lv et al. [27]; (**i**) The method of Wu et al. [35]; (**j**) The proposed method.

The qualitative comparison of the backlit images is displayed in Figure 6. We selected six degraded backlit images from the test set that suffer from different kinds of low contrast. The image quality is assessed primarily through human visual perception, especially on the color, the contrast, and the sharpness. From a global perspective, eight comparison methods all work well for removing the backlit effect. Depending on the details of the processing results, we divided the results of the eight comparison methods into four categories from visual perception. The first category is the results of Li et al. [40]; we can observe a notable enhancement in both brightness and contrast in the mountain within the backlit region. However, the backlit effect is not fully removed in the area around the edge of the mountain body, and black artifacts are introduced in the middle parts of the sky. This is because the optimized strategy in the method by Li et al. [40] use the local patch of the image to set the filter parameters, which results in an increase in brightness for dark areas and a decrease in brightness for bright areas. The second category includes the results of Fu et al. [7] and Wang et al. [8]; both approaches involve the decomposition of an image into reflectance and illumination components, aiming to retain both the fine details and the natural appearance of the image. Nevertheless, there is a slight haze effect in the processed images using both the methods of Fu et al. [7] and Wang et al. [8]. Meanwhile, we found that the method of Fu et al. [7] shows limited capability in processing severely backlit images compared to the method of Wang et al. [8]; this is due to the adoption of a bi-log transformation in Wang et al.’s method [8], which is employed to map the illumination and strike a balance between preserving details and achieving a natural appearance. The third category consists of the results of Shi et al. [6] and Zhang et al. [41]; both methods can remove the haze and the backlit effect, and the results are close to a black-and-white image style, especially when using the method of [41]. There is a similar limitation to the second category that the method of Shi et al. [6] is not as good as the method of Fu et al. [7] for addressing severely degraded backlit images. Although the results in the third category show good performance in restoring the backlit and haze effects, there is a lack of realistic color information. The fourth category results are generated from the methods of Ying et al. [36], Lv et al. [27], and Wu et al. [35]. The haze appearances in the results are very similar to the ones in the results of deep learning-based methods [27,35]. Additionally, they have limited capability to refine the fading of color intensity as light travels through the atmosphere. Nevertheless, they excel in terms of maintaining consistent brightness and contrast recovery across images with varying degrees of backlighting. Considering the findings presented in Figure 6 and the analysis provided earlier, we have verified that the proposed method attains the highest level of human visual perception with regard to color, contrast, and brightness.

### 4.3. Quantitative Evaluation

**Figure 7:**
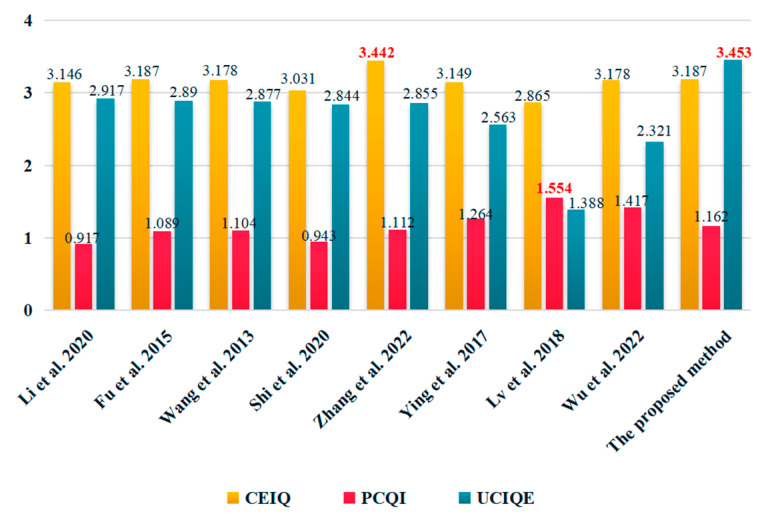
The average trend of various comparison methods assessed quantitatively, and the results of the comparison methods in the figure are from [6,7,8,27,35,36,40,41] and the proposed method. The UCIQE values have been scaled down by a factor of ten, and the bold values indicate the superior results.

**Figure 8:**
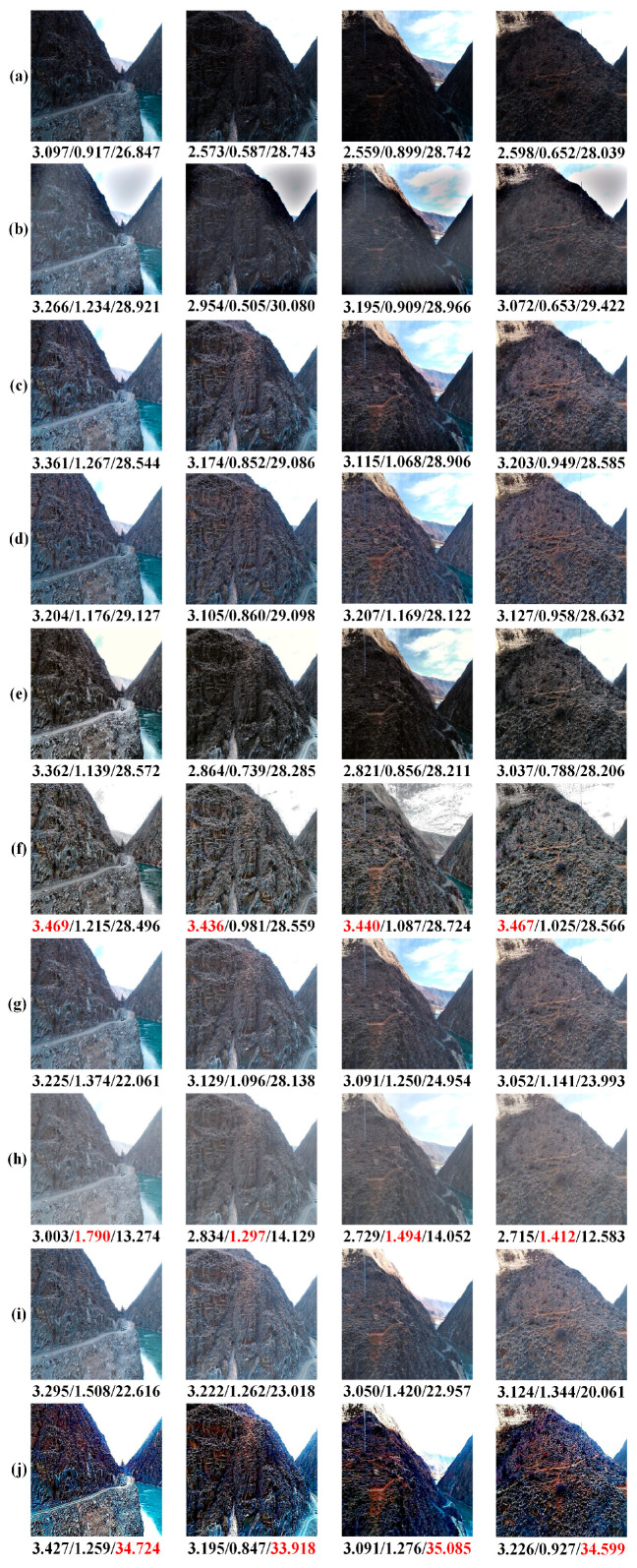
Quantitative outcomes for a single image using CEIQ, PCQI, and UCIQE; the highest score is in red. (**a**) The input images; (**b**) The method of Li et al. [40]; (**c**) The method of Fu et al. [7]; (**d**) The method of Wang et al. [8]; (**e**) The method of Shi et al. [6]; (**f**) The method of Zhang et al. [41]; (**g**) The method of Ying et al. [36]; (**h**) The method of Lv et al. [27]; (**i**) The method of Wu et al. [35]; (**j**) The proposed method.

The experimental results are presented in Figure 7, which displays the average values of three evaluation metrics, CEIQ, PCQI, and UCIQE. The comparison methods were measured quantitatively using the test dataset, allowing for a comprehensive assessment of their effectiveness. The quantitative result of Li et al. [40] is not stable; it obtains a competitive score on the UCIQE metric but shows poor performance on both CEIQ and PCQI metrics. As for the quantitative results of Fu et al. [7] and Wang et al. [8], they show good performance and there are very small gaps in the scores of the three evaluation metrics. Although the methods of Shi et al. [6] and Zhang et al. [41] generate images with similar styles during qualitative analysis, the quantitative result of Zhang et al. [41] is much better than the quantitative result of Shi et al. [6]. This aligns with the qualitative analysis, indicating that the approach described by Shi et al. [6] demonstrates limited effectiveness in restoring heavily degraded backlit images. Regarding the quantitative results of Ying et al. [36], Lv et al. [27], and Wu et al. [35], they do not perform well on the scores of CEIQ and UCIQE but obtain a higher PCQI score. After comparing it with the other eight methods on the scores of CEIQ, PCQI, and UCIQE, the proposed method secures the second, fourth, and first positions, respectively. It can effectively enhance severely degraded backlit images, achieving outstanding visual quality and competitive quantitative scores. Moreover, we selected four images from the test set to display the visual effect and the quantitative results of a single image on three evaluation metrics (as shown in Figure 8). Based on Figure 8, it is evident that the quantitative analysis of individual images aligns with the results obtained from the mean assessment in Figure 7. Although the proposed method does not achieve the first position in terms of the CEIQ and PCQI evaluation metrics, it is still ranked in the top four of the comparative methods. Researchers usually judge the effectiveness of an algorithm by combining the results of both qualitative and quantitative results. Therefore, the proposed method shows a more satisfying performance than the other comparison methods, and it is robust when processing different levels of backlit images.

### 4.4. Application Test

**Figure 9:**
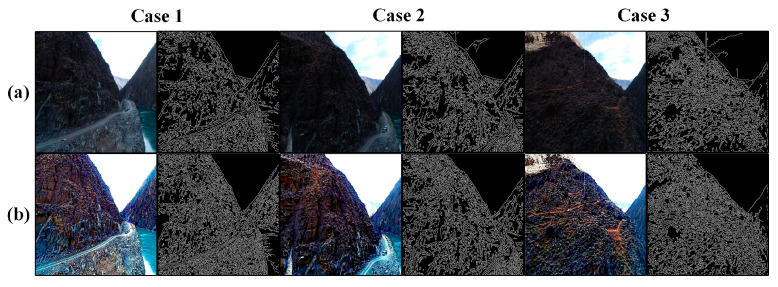
Edge detection application test using Canny. (**a**) The original images; (**b**) The enhanced images (processed using the proposed method).

**Figure 10:**
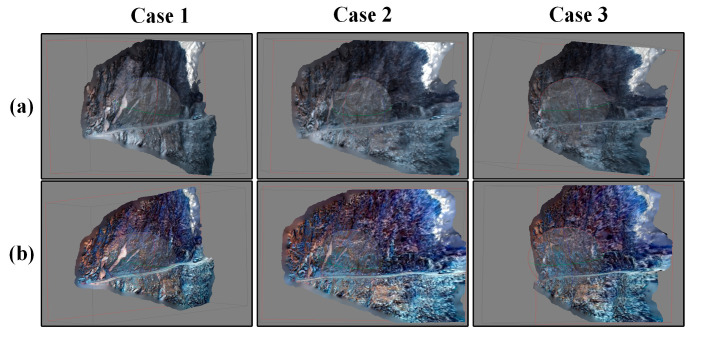
The 3D reconstruction application test. (**a**) The results of 3D reconstruction using the original images; (**b**) The results of 3D reconstruction using the enhanced images (processed using the proposed method).

**Figure 11:**
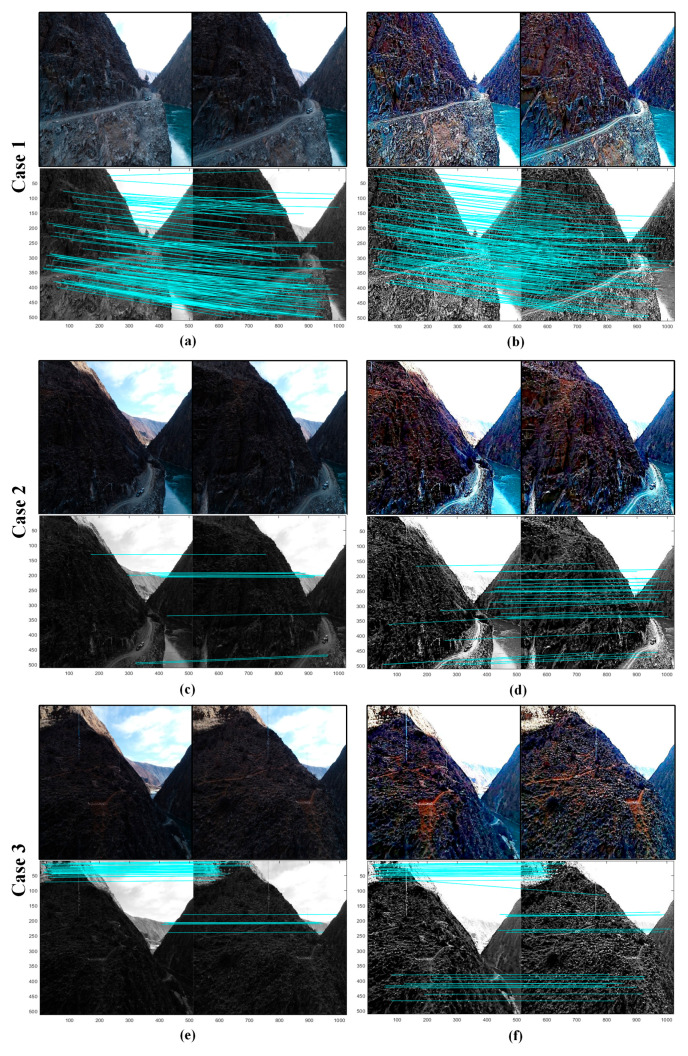
Application test using SIFT. (**a**,**c**,**e**) The original images from cases 1, 2, and 3; (**b**,**d**,**f**) The enhanced results (processed using the proposed method).

We assessed the effectiveness of the proposed method through real-world computer vision applications, including 3D reconstruction and image feature extraction. More specifically, we used Canny edge detection [52] and scale-invariant feature transform (SIFT) operators [53]. The test codes of Canny edge detection and SIFT were downloaded from github, and the 3D reconstruction application was achieved through Agisoft (Agisoft PhotoScan Pro) [54]. To demonstrate the performance of these applications, we conducted tests on both the original images and the enhanced images. The experimental results of Canny edge detection and 3D reconstruction are shown in Figure 9 and Figure 10, which are illustrated in a qualitative way. One can notice that during edge feature extraction, the enhanced images reveal more details in comparison to the original images. We also found that the 3D model constructed using the enhanced images is much clearer and more vivid than the model constructed using the original images. Regarding the SIFT operator, the relevant experimental outcomes are shown in Figure 11 and Table 3. It is evident from these results that the proposed method substantially boosts both the quantity of key points and the matching pairs.

## 5. Conclusions

To address the degraded backlit images captured using a UAV on a high-steep slope, we proposed a novel image restoration framework, utilizing both the Retinex theory model and the physical imaging formation model to its benefit. We took the real-world application into account and also addressed the color distortion issue in the backlit images captured in the high-steep slope scenes. We first employed the Retinex theory model and backlight removal strategy to eliminate the backlit effect, and then the image color and details were further enhanced using the physical imaging formation model. Both qualitative and quantitative results affirm that the proposed approach outperforms state-of-the-art methods in restoring deteriorated backlit images sourced from a real-world dataset captured on the steep left bank of the Sequ Bridge. Moreover, we employed the enhanced images to explore several applications including edge detection, 3D reconstruction, and feature matching. The results indicate that the proposed method holds significant promise for practical, real-world applications.

Despite the satisfying performance, the proposed method nevertheless has some limitations. The rock color details of the geological environment in the restored image were not adequately considered, which could lead to errors in subsequent geologists’ judgments of the engineering geological conditions in the 3D model. Additionally, the problem of local shadows in UAV images in high-steep slope scenes has not yet been studied. In the future, efforts will be made to address the aforementioned issues.

## Figures and Tables

**Table 1:** The parameters of the UAV platform and the on-board camera.

The UAV Platform	
**Weight (kg)**	6.3
**Size (mm)**	810 × 670 × 430
**Max Lifting Speed (m/s)**	6
**Max Horizontal Speed (m/s)**	23
**Max Altitude (m)**	5000
**Max Range Time (min)**	55
**RTK Position Accuracy**	1 cm + 1 ppm (horizontal) 1.5 cm + 1 ppm (perpendicular)
**The On-Board Camera**	
**Weight (g)**	800
**Size (mm)**	198 × 166 × 129
**Sensors**	Size (mm): 35.9 × 24
	Effective Pixels (million): 45
	Picture Element Size (μm): 4.4

**Table 2:** Quantitative results from the ablation study, where the values represent the average across testing examples ^a,b,c^.

Methods and Metrics	CEIQ	PCQI	UCIQE
**Backlit removal**	3.127(2)	**1.379(1)**	27.728(3)
**Color and detail enhancement**	2.267(3)	0.850(3)	31.791(2)
**The proposed method**	**3.187(1)**	1.162(2)	**34.534(1)**

^a^ Image non-reference-quality metrics, namely CEIQ, PCQI, and UCIQE, are used for comparison. ^b^ The values highlighted in bold indicate the top-performing outcomes. ^c^ The number within parentheses indicates the method’s ranking on the metric, ranging from 1 to 3.

**Table 3:** The number of key points and matching for local feature point matching ^a^.

Test Data		Number of Key Points		Number of Matching
		Left	Right	
**Typical example from Case 1**	**Original**	2117	1150	186
	**Enhanced**	**3639**	**3587**	**188**
**Typical example from Case 2**	**Original**	639	579	11
	**Enhanced**	**3968**	**4342**	**31**
**Typical example from Case 3**	**Original**	547	935	42
	**Enhanced**	**4670**	**5362**	**46**

^a^ The values highlighted in bold indicate the top-performing outcomes.

## Data Availability

Data underlying the results presented in this paper are not publicly available at this time but may be obtained from the author upon reasonable request.
